# The Protective Effect of Fucoidan in Rats with Streptozotocin-Induced Diabetic Nephropathy

**DOI:** 10.3390/md12063292

**Published:** 2014-05-30

**Authors:** Jing Wang, Huaide Liu, Ning Li, Quanbin Zhang, Hong Zhang

**Affiliations:** 1 Institute of Oceanology, Chinese Academy of Sciences, Qingdao 266071, China; E-Mails: jingwang@qdio.ac.cn (J.W.); wangjing_17@163.com (Q.Z.); hongzhang@qdio.ac.cn (H.Z.); 2 School of Life Sciences, Nantong University, Seyuan Road 9, Nantong 226019, China; E-Mail: hold126@126.com; 3 Nantong Branch, Institute of Oceanology, Chinese Academy of Sciences, Nantong, Jiangsu 226006, China; 4 Collaborative Innovation Center for Marine Biomass Fibers, Materials and Textiles of Shandong Province, Qingdao 266071, China

**Keywords:** sulfated polysaccharide, fucoidan, diabetic nephropathy

## Abstract

Diabetic nephropathy (DN) has long been recognized as the leading cause of end-stage renal disease, but the efficacy of available strategies for the prevention of DN remains poor. The aim of this study was to investigate the possible beneficial effects of fucoidan (FPS) in streptozotocin (STZ)-induced diabetes in rats. Wistar rats were made diabetic by injection of STZ after removal of the right kidney. FPS was administered to these diabetic rats for 10 weeks. Body weight, physical activity, renal function, and renal morphometry were measured after 10 weeks of treatment. In the FPS-treated group, the levels of blood glucose, BUN, Ccr and Ucr decreased significantly, and microalbumin, serum insulin and the β2-MG content increased significantly. Moreover, the FPS-treated group showed improvements in renal morphometry. In summary, FPS can ameliorate the metabolic abnormalities of diabetic rats and delay the progression of diabetic renal complications.

## 1. Introduction

Diabetic nephropathy (DN) is a major complication of diabetes and a leading cause of end-stage renal failure throughout much of the world [1]. DN is characterized by changes in both glomerular and tubular structure and function. The pathogenesis of DN includes genetic, hemodynamic and metabolic factors, and oxidative stress as well as renal hypertrophy, but the exact mechanism is not clear [2]. Most studies have focused on alterations in the glomerulus, including abnormalities in glomerular permeability and capillary pressure, glomerular hyperplasia or hypertrophy and increases in mesangial volume [3,4]. To prevent and treat diabetic nephropathy, current methods using agents such as angiotensin-converting enzyme inhibitors, angiotensin-receptor blockers and antihypertensive drugs have been tried in clinical practice [5]. Unfortunately, currently available medical interventions are unable to effectively reverse or even delay the progression of DN [6]. 

Polysaccharides can reduce blood glucose levels in normal rats, streptozotocin (STZ)-induced diabetic rats and alloxan-induced diabetic rats [7]. He *et al.* found that *Ganoderma lucidum* polysaccharide could ameliorate the metabolic abnormalities of diabetic mice and prevent or delay the progression of diabetic renal complications [8]. Zhang *et al.* found that *Astragalus* polysaccharide improved early diabetic nephropathy and affected the mRNA expression of NF-κB and IκB in the renal cortex of STZ-induced diabetic rats [9].

Sulfated polysaccharides are considered to be an attractive class of compounds as drug candidates [10,11]. Fucoidans are highly sulfated cell-wall polysaccharides found mainly in various species of brown seaweeds such as *Saccharina japonica*, *Undaria pinnatifida*, and *Sargassum* C. Ag., and variant forms of fucoidans have also been found in animal species, including the sea cucumber [12]. Fucoidan has been reported to possess diverse biological activities of potential medicinal value, such as anticoagulant, antitumor, anti-inflammatory, antiviral and antioxidant activities [13]. 

The brown seaweed, *Saccharina japonica*, is a common seafood in China and many other countries, and has been documented as a drug in traditional Chinese medicine for over a thousand years. In the past thousand years and more, Chinese people have been using it as a traditional medicine to cure edema disease, a symptom of kidney disease [14]. FPS extracted from *Saccharina japonica* is an acidic sulfated polysaccharide, mainly made of fucose, galactose and sulfate, with smaller amounts of mannoses, glucuronic acid, glucose, rhamnose, arabinose and xylose. Recent studies have corroborated a renoprotective role for fucoidan in animal models of kidney injury. Our previous studies have found that FPS and its derivatives extracted from *Saccharina japonica* are effective in ameliorating abnormal biochemical changes in experimental chronic renal failure (CRF) [15]. The mechanism of action of FPS derivatives in CRF rats is related to the antioxidant activities, the substituted group and the molecular weight of FPS [16]. Zhang *et al.* also found that FPS could inhibit the development of proteinuria associated with Heymann nephritis [17,18]. Furthermore, we found that FPS exhibited a considerable hypoglycemic effect, possibly by stimulating the pancreatic release of insulin and/or by reducing insulin metabolism [19]. Diabetic retinopathy (DR) is one of the severe complications of diabetes. Our study found that low molecular weight fucoidan could alleviate diabetic retinal neovascularization and damage, likely through lowering HIF-1α and VEGF expressions [20]. Diabetic patients are at high risk of endothelial and vascular dysfunction, we also demonstrated that fucoidan could protect vasoendothelial function and reduce basal blood pressure in type 2 diabetes rats via, at least in part, preservation of endothelial NO synthase (eNOS) function [21].

Oxidative stress has been considered to be a common pathogenic factor in DM and its complications including nephropathy. In DN, free radicals have been shown to decrease *de novo* synthesis of heparin sulfate proteoglycans, which correlates with proteinuria seen in this condition [22]. Ha *et al.* demonstrated increased 8-hydroxydeoxyguanosine (8-OHdG) in STZ-induced diabetic kidneys and suggest that formation of 8-OHdG and, therefore, oxidative damage are closely related in the process of diabetic nephropathy [23]. Brezniceanu *et al.* demonstrated that renal catalase overexpression in db/db mice attenuated reactive oxygen species (ROS) generation, angiotensinogen, proapoptotic gene expression and apoptosis in the kidneys of diabetic mice *in vivo*. This study points to an important role of ROS in the pathophysiology of diabetic nephropathy [24]. Combination of strategies to prevent overproduction of ROS and to increase the removal of performed ROS may prove to be effective in preventing the development and progression of diabetic nephropathy [25]. Zhang *et al.* found that *Astragalus membranaceus* (root) as a free radical scavenger implies its effect against oxidative stress in the early stages of DN [6]. Bhatia *et al.* evaluate the oxidative stress status in Asian Indian patients suffering from type 2 diabetes mellitus (DM) with nephropathy. Results of their study indicate that oxidative stress is increased and antioxidant defenses are compromised in type 2 DM. These derangements are of a higher magnitude in patients of type 2 DM with nephropathy [26].

Our previous study found that FPS had antioxidant activity, renoprotective and hypoglycemic activity *in vivo* and *in vitro* [15,19,27]. Thus there is a need to study whether FPS has renal protective effects in STZ-induced diabetic rats. 

## 2. Results and Discussion

### 2.1. Results

#### 2.1.1. Chemical Analysis

The chemical composition of the FPS was analyzed in this study. The results showed that the principal chemical components of the FPS were fucose and sulfate along with uronic acid and a small amount of protein. The fucose and sulfate content was 29.12% and 33.01%, respectively. The constituents of the neutral monosaccharide of the FPS were analyzed with high performance liquid chromatography (HPLC). The results showed that fucose was the main form of sugar, representing 62.08% of the total neutral sugar in the FPS. In addition to fucose and galactose, mannose, glucose, xylose, and arabinose were also observed in the FPS. The molecular weight of FPS was 87,000 Da. These results show that the chemical properties of FPS may substantially influence its activity, as demonstrated in this study.

#### 2.1.2. Effects of FPS on Physical Activity

**Figure 1:**
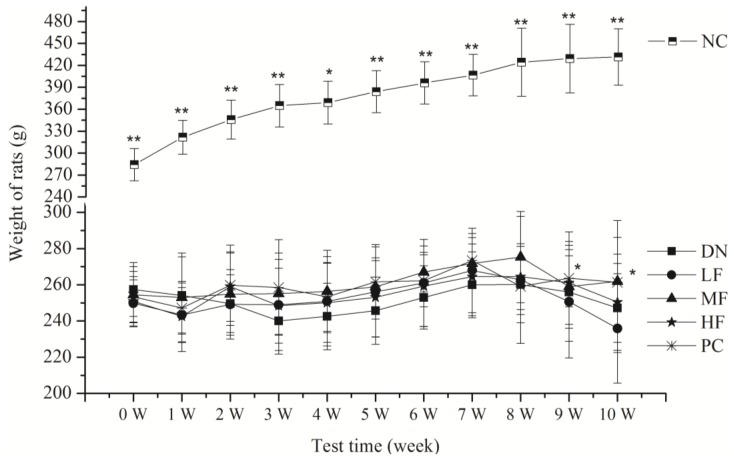
Effects of fucoidan (FPS) on the weight of streptozotocin (STZ)-induced diabetic nephropathy rat. NC: Normal control group; DN: Diabetic nephropathy group; LF: FPS, 75 mg/kg body wt; MF: FPS, 150 mg/kg body wt; HF: FPS, 300 mg/kg body wt; * *p* < 0.05, ** *p* < 0.01, compared with the DN group. Statistical analysis was performed using ANOVA. Data are presented as mean ± S.D.

All the rats in the normal control group (NC) group survived and exhibited normal physical appearance and behavior during the experiments, showing smooth fur and weight gain (Figure 1). However, two rats died in the other groups. The surviving rats in the DN group exhibited unresponsive behavior and rough fur and were significantly thinner during the experiments. Their eyes showed a 7/20 ratio of cataracts. The surviving rats in the high fucoidan (HF), medium fucoidan (MF) and PC groups exhibited significantly better behavior with little rough fur and a ratio of cataracts of 3/20, 4/20 and 4/20, respectively. However, the surviving rats in the low fucoidan (LF) group exhibited no significant changes compared with the DN group. The ratio of cataracts in the LF group was 3/20. The weight of the rats in all groups except the NC group showed no significant changes compared with the DN group.

#### 2.1.3. Effects of FPS on Blood Glucose

**Figure 2:**
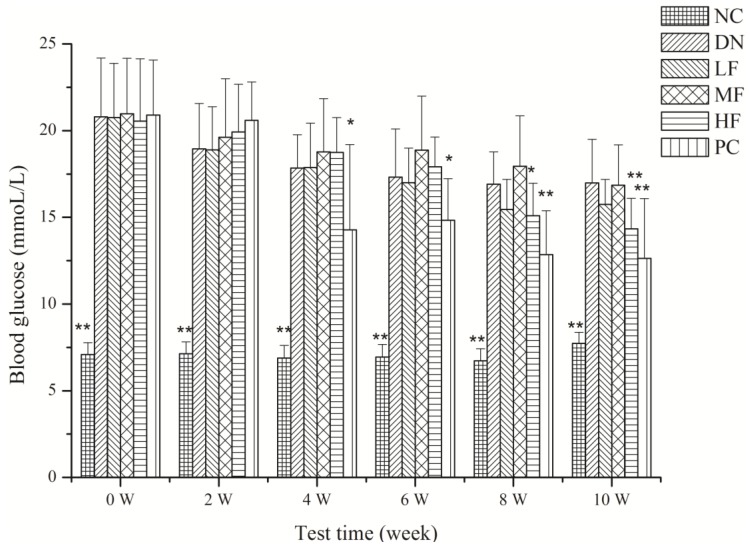
Effects of FPS on the weight of STZ-induced diabetic nephropathy rat. NC: Normal control group; DN: Diabetic nephropathy group; LF: FPS, 75 mg/kg body wt; MF: FPS, 150 mg/kg body wt; HF: FPS, 300 mg/kg body wt; * *p* < 0.05, ** *p* < 0.01, compared with the DN group. Statistical analysis was performed using ANOVA. Data are presented as mean ± S.D.

The effects of FPS on blood glucose in the rats with STZ-induced DN are shown in Figure 2. The blood glucose level increased significantly in the DN and other treatment groups relative to the NC group at week 0 and at the 2nd, 4th, 6th, 8th and 10th weeks (Figure 2). The HF group showed a significantly decreased blood glucose level after 8 weeks compared with the DN group. The blood glucose level decreased significantly in the PC group at 4, 6, 8 and 10 weeks. The MF and LF treatment group showed no significant effect on blood glucose. 

#### 2.1.4. Effects of FPS on Renal Function

**Figure 3:**
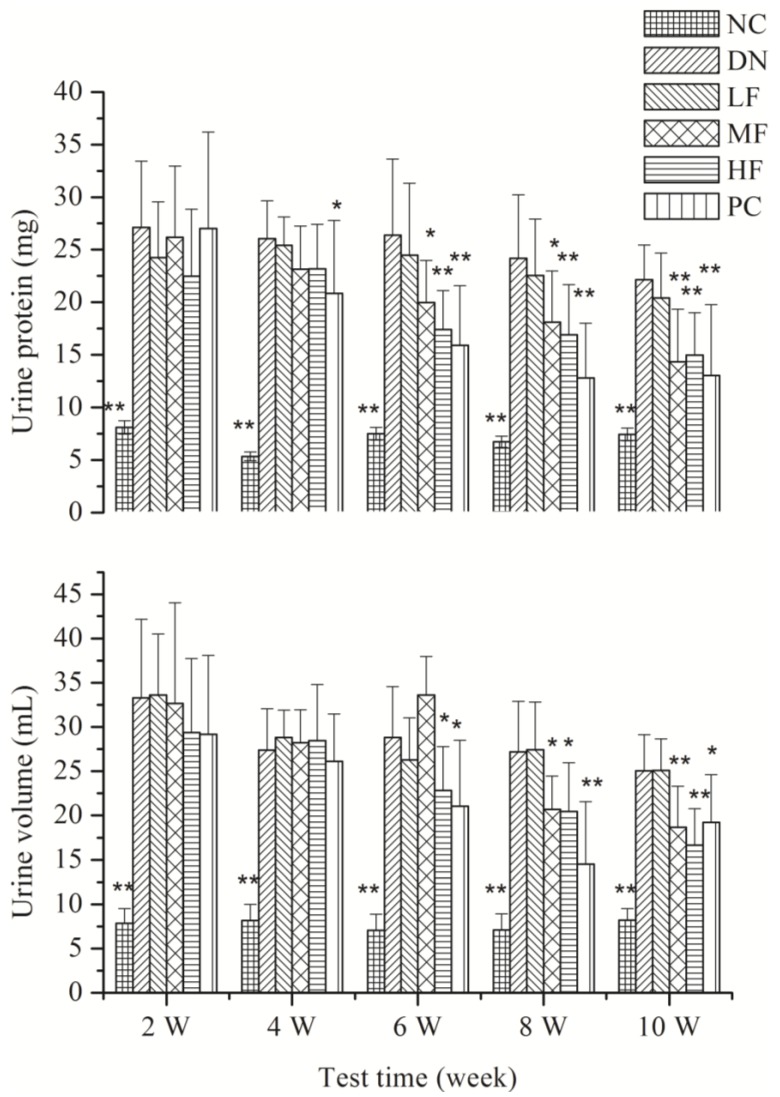
Effects of FPS on the weight of STZ-induced diabetic nephropathy rat. NC: Normal control group; DN: Diabetic nephropathy group; LF: FPS, 75 mg/kg body wt; MF: FPS, 150 mg/kg body wt; HF: FPS, 300 mg/kg body wt; * *p* < 0.05, ** *p* < 0.01, compared with the DN group. Statistical analysis was performed using ANOVA. Data are presented as mean ± S.D.

Figure 3 shows the effects of FPS on urine volume and urine protein in the rats with STZ-induced DN. Compared with the NC group, the 24 h urine volume and urine protein increased significantly in the other groups at 2, 4, 6, 8 and 10 weeks. The HF group showed a significant decrease in 24 h urine volume and urine protein at 6, 8 and 10 weeks compared with the DN group.

The effects of FPS on the ratio of kidney weight to body weight, renal function, serum insulin, glycosylated hemoglobin, microalbumin and β2-MG in the rats with STZ-induced DN are shown in Table 1. Compared with the NC group, the rats in the DN group and treatment group showed renal hypertrophy and a significant increase in relative kidney weight. However, the HF and PC groups showed a significant decrease in the ratio of kidney weight to body weight.

BUN increased significantly in the DN group compared with the NC group and decreased significantly in the MF and HF groups. However, the treatment group had no significant effect on Scr. The HF and PC groups showed higher effects on urinary creatinine excretion (Ucr) and creatinine clearance (Ccr). Serum insulin, glycosylated hemoglobin and β2-MG decreased significantly in the DN group compared with the NC group. The HF group showed an increased insulin and microalbumin level compared with the DN group. All treatment groups showed an increased level of β2-MG. 

#### 2.1.5. Effects of FPS on Renal Morphological Changes

**Figure 4:**
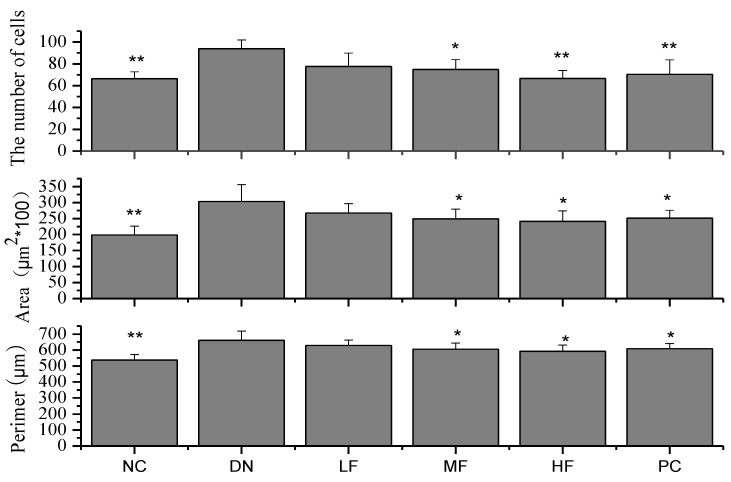
Effects of FPS on the weight of STZ-induced diabetic nephropathy rat. NC: Normal control group; DN: Diabetic nephropathy group; LF: FPS, 75 mg/kg body wt; MF: FPS, 150 mg/kg body wt; HF: FPS, 300 mg/kg body wt; * *p* < 0.05, ** *p* < 0.01, compared with the DN group. Statistical analysis was performed using ANOVA. Data are presented as mean ± S.D.

**Figure 5:**
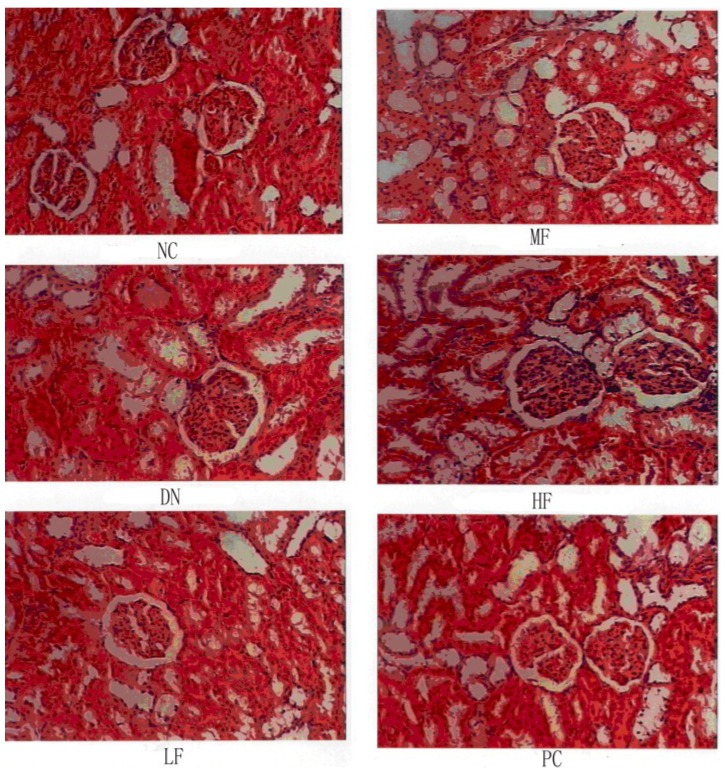
Effects of FPS on the weight of STZ-induced diabetic nephropathy rat. NC: Normal control group; DN: Diabetic nephropathy group; LF: FPS, 75 mg/kg body wt; MF: FPS, 150 mg/kg body wt; HF: FPS, 300 mg/kg body wt; * *p* < 0.05, ** *p* < 0.01, compared with the DN group. Statistical analysis was performed using ANOVA. Data are presented as mean ± S.D.

The NC group showed no changes in kidney tissue morphology, whereas the DN group showed significant changes in kidney tissue morphology (Figure 4 and Figure 5). The glomerular volume and the number of cells increased, reflecting the dilation of glomerular capillaries and the tubular epithelial cell degeneration in the rats of the DN group. The morphological changes occurring in the kidney varied among treatment groups. Compared with the DN group, the MF and HF groups showed significantly decreased glomerular capsule perimeters, cross-sectional areas and cell numbers.

**Table 1:** Effect of FPS on the ratio of the weight of the kidney to body weight, renal function, serum insulin, glycosylated hemoglobin, microalbumin and β2-microglobulin in the STZ-induced diabetic nephropathy rats.

Group	Relative kidney weight (%)	BUN (μg/mL)	Scr (μmol/L)	Ucr (μmol/L)	Ccr (mL/min)	Serum insulin (μIU/mL)	Glycosylated hemoglobin (%)	Microalbumin (μg/mL)	β2-MG (μg/mL)
NC	0.31 ± 0.05 **	6.44 ± 0.65 **	42.69 ± 3.54	11,968.4 ± 978.7 **	1.61 ± 0.32 **	10.83 ± 2.92 **	21.16 ± 6.41 **	1.42 ± 0.15 *	0.12 ± 0.04 **
DN	0.82 ± 0.12	25.40 ± 7.90	40.18 ± 4.81	2359.5 ± 490.4	1.00 ± 0.15	5.74 ± 1.43	18.28 ± 1.66	1.25 ± 0.15	0.07 ± 0.05
LF	0.78 ± 0.11	21.55 ± 6.29	39.87 ± 3.33	2703.4 ± 312.4	1.19 ± 0.25	5.87 ± 1.33	19.52 ± 2.08	1.33 ± 0.18	0.11 ± 0.03 *
MF	0.73 ± 0.13	18.48 ± 6.99 *	42.27 ± 5.42	4476.5 ± 1238.1 **	1.35 ± 0.41	6.67 ± 1.38 *	17.29 ± 2.59	1.47 ± 0.29	0.08 ± 0.03
HF	0.70 ± 0.06 *	18.42 ± 5.13 *	44.11 ± 5.05	5289.1 ± 1607.1 **	1.40 ± 0.42 *	6.18 ± 1.34 *	18.78 ± 2.89	1.42 ± 0.18 *	0.11 ± 0.04 *
PC	0.70 ± 0.10 *	18.56 ± 5.49 *	41.40 ± 4.90	4671.0 ± 1274.4 **	1.46 ± 0.43 *	6.61 ± 1.53 *	17.32 ± 4.53	1.45 ± 0.18 *	0.09 ± 0.04

NC: Normal control group; DN: Diabetic nephropathy group; LF: FPS, 75 mg/kg body wt; MF: FPS, 150 mg/kg body wt; HF: FPS, 300 mg/kg body wt; BUN: blood urea nitrogen; Scr: serum creatinine; Ucr: urine creatinine; Ccr: creatinine clearance; β2-MG: β2-microglobulin; * *p* < 0.05, ** *p* < 0.01, compared with the DN group. Statistical analysis was performed using ANOVA. Data are presented as mean ± S.D.

### 2.2. Discussion

The kidneys are vital for removing toxic waste products from the body and for maintaining fluids, minerals, and electrolytes at physiological levels. Elevated blood glucose can damage the cells and microblood vessels of the kidney [28]. Our previous study found that FPS affected chronic renal failure and diabetic complications [15,22]. However, very little is known about the preventive or therapeutic effect of FPS against diabetic complications, including nephropathy. It is important to examine the effectiveness of herbal drugs on these diseases from the standpoint of the development of new bioactive resources. STZ can selectively damage insulin-producing pancreatic endocrine cells and induce experimental hyperglycemia. These effects are stable and long lasting. The extraction of one kidney can increase the burden on the contralateral kidney and accelerate the progression of the disease, accelerating the development of DN. 

Both decreases and gains in body weight were observed in conjunction with the progression of diabetes. The inhibition of these decreases in body weight has been viewed as evidence of the successful treatment of diabetes and diabetic complications [29]. In this study, the FPS dose-dependently inhibited the loss and/or gain in body weight relative to the DN group. 

High blood glucose is known to be an important cause of DN. The control of glucose can prevent the occurrence and development of DN. Specifically, the control of blood glucose is one of the important measures used for the prevention and treatment of DN [30]. The results of the current study showed that the blood glucose in the rats of the DN group increased significantly and that this increase persisted for 10 weeks. The HF group showed a marked hypoglycemic effect after the administration of FPS for 8 and 10 weeks. We hypothesize that HF can prevent and treat DN by reducing the blood glucose level.

During the progression of diabetes, an increase in kidney weight, the elevation of the BUN and Scr levels due to interstitial atrophy and vasodilated atrophic changes in the glomeruli and tubules, including epithelial necrosis and ballooning with focal fibrosis, known as diabetic nephropathies, are generally observed [31]. In addition, these indices have been used to observe the occurrence and progression of DN [32]. An improvement in these abnormal changes is considered direct evidence of an improvement in DN [33]. In this study, the 24 h urine protein and urine volume increased significantly two weeks after the initiation of the disease model. Ten weeks after the initiation of the disease model, renal hypertrophy was observed, renal function was abnormal and BUN increased significantly. These observations showed that the model of DN had been successfully established. The DN rats showed damage to the kidney and renal insufficiency. As a result of these effects, BUN increased and Ucr and Ccr decreased significantly. Accordingly, many toxic substances accumulated in the body and aggravated the DN. The experimental results reported in this study showed that FPS could significantly decrease the BUN content and increase Ucr and Ccr levels, producing significant improvements in renal function. This outcome means that FPS effectively controls DN.

Renal hypertrophy is an important pathological feature of early DN, and the control of kidney hypertrophy is an important sign of the effectiveness of drugs used to treat DN. The experimental results reported in this study showed that FPS could significantly decrease the kidney coefficient of DN rats and inhibit renal hypertrophy. These results also showed that FPS could diminish glomerular damage in diabetic rats. 

Recent studies strongly support the concept that the primary cause of DN involves a metabolic disorder. In particular, the importance of hyperglycemia as a risk factor for DN is supported by several observations and pieces of experimental evidence [34]. In this study, all treatment groups showed an elevated β2-MG level and certain treatment groups showed increased levels of serum insulin and glycosylated hemoglobin. These results suggest that the mechanism of renal protection by FPS is, most likely, the modulation of metabolic abnormalities in the hyperglycemic state. Additionally, FPS had obvious hypoglycemic effects, as previously demonstrated. In this study, hypoglycemic effects were also identified as a possible reason for the improvement of nephropathy. Therefore, further studies are planned to determine the mechanisms of action of FPS on diabetes and DN.

Increasing evidence in both experimental and clinical studies suggests that oxidative stress plays a major role in the pathogenesis of both types of diabetes mellitus. Oxidative stress is increased in diabetes, and the overproduction of ROS in diabetes is a direct consequence of hyperglycemia. Abnormally high levels of free radicals and the simultaneous decline of antioxidant defense mechanisms can lead to damage of cellular organelles and enzymes, increased lipid peroxidation, and development of insulin resistance [19]. Many researchers suggest that antioxidants may be helpful in the treatment of DN [25]. *Lycium barbarum* polysaccharide (LBP) has been shown to have hypoglycemic and diabetic nephropathy properties in a streptozotocin-induced diabetic rat model. Diabetic rats treated with LBP-4 (10 mg/kg) for 8 weeks showed increased activity of antioxidant enzymes and increased scavenging of oxygen radicals, while the activity of protein kinase C (PKC) in the renal cortex was maintained at a physiological level [34]. *Ganoderma lucidum* polysaccharides (GL-PS) can positively influence the metabolic abnormalities of diabetic mice and prevent or delay the progression of diabetic renal complications, and the mechanism is related to its antioxidant activity [35]. Our previous study found that fucoidan and its fractions had strong antioxidant activity *in vitro* and *in vivo*, the mechanism of fucoidan on chronic kidney disease (CKD) rats was related to their antioxidant activities, the samples of which could enhance the activity of antioxidant enzymes and reduce the LPO level, which alleviated the symptom of CKD complications [15,31]. We suppose the mechanism of fucoidan on the DN rats also was related with its antioxidant activity.

Diabetic nephropathy (DN) is the major life-threatening complication of diabetes. Abnormal permselectivity of the glomerular basement membrane (GBM) plays an important role in DN pathogenesis. Heparanase is the predominant enzyme that degrades heparan sulfate (HS), the main polysaccharide of the GBM. Gil *et al.* found the crucial role of heparanase in the pathogenesis of DN and its potential as a highly relevant target for interventions in patients with DN [36]. The chemical characteristics of fucoidan and HS are that both of them have sulfated group, a carbohydrate chain and a negative charge, we suppose fucoidan could react with heparanase in DN rats in order to reduce the lack of HS. However, the exact mechanism needs further study.

## 3. Experimental Section

### 3.1. Materials

*Saccharina japonica*, cultured in Rongcheng, China, was collected in July 2012. The fresh algae were promptly washed, sun dried, and kept in plastic bags at room temperature for use. 

### 3.2. Preparation of Natural Polysaccharides

FPS was extracted according to the modified method of Wang *et al.* [27]. Generally, 100 g dry algae were cut roughly and autoclaved in water at 115–125 °C for 3 h. The hot aqueous solution was separated, concentrated, dialyzed, precipitated, and then dried to give polysaccharide, named FPS (yield 2.3%).

### 3.3. Animals

One hundred thirty male wistar rats were obtained from the Institute of Zoology, Chinese Academy of Medical Sciences. The feeding and care of the animals followed the Guiding Principles for Care and Use of Laboratory Animals of China. The animals were housed in a controlled environment (at a temperature of 24 ± 1 °C and under a 12 L:12 D lighting cycle with the light turned on at 7 a.m.) and were given free access to standard rat food and water but were not treated with insulin or any other anti-diabetic drugs. This project was approved by Chinese Academy of Medical Sciences on January 2013. The project identification code was 20130106.

### 3.4. Experimental Protocols

Two steps were used to develop the DN model. First, the rats received intraperitoneal anesthesia with 40 mg/kg sodium pentobarbital. An incision was made in the skin of the back. The muscle was separated, the right kidney was removed and the muscle and skin were sutured. The rats then received conventional care for 2 weeks. Ten additional rats were assigned to a sham operation group. The operation was performed without removal of the right kidney. This group served as the control group (NC group). Second, the experimental rats received 50 mg/kg of STZ (freshly dissolved in 0.1 mmol/L citrate buffer, pH 4.5) intraperitoneally after fasting for 12 h [9]. The NC group received only the same volume of citrate buffer. Two days after the STZ treatment, the development of diabetes in the experimental rats was confirmed by measuring the levels of glucose in the blood (sampled from the tail vein) and urine. Rats with blood glucose levels of 17 mmol/L or higher and strongly positive urine glucose levels were considered diabetic. The blood glucose levels in the NC group remained normal for the duration of the study. A total of 60 rats that developed DN was randomly assigned to five groups of 12 rats each: the DN model group (DN group); the DN rats treated with FPS (*i.e.*, the HF, MF and LF groups); and the DN positive control group (PC group). The rats were gavaged as follows: 10 mL/kg/day 0.5% CMC for the NC and DN groups; 300 mg/kg, 150 mg/kg and 75 mg/kg FPS for the HF, MF and LF groups, respectively; and 15 mg/kg gliguidone and 1.5 mg/kg lotensin for the PC group. All groups were gavaged once daily at the same time for 10 weeks. 

### 3.5. Biochemical Analysis

The total sugar content of FPS was determined according to the method of Dubois *et al.* using l-fucose as the standard [37]. Sulfate content was analyzed with the barium chloride-gelatin method of Kawai *et al.* [38]. Uronic acid was estimated in a modified carbazole method using d-glucuronic acid as the standard [39]. Neutral sugar composition was determined with HPLC chromatography [40]. The molecular weight of the sample was assayed by a high performance—gel permeation chromatography (HP-GPC) system at 40 °C, where 2.84% Na_2_SO_4_ solution was used as mobile phase with a flow rate of 0.5 mL/min. TSK G300 column (300 mm × 7.8 mm) and 2140 refractive index detector was used. A series of different molecular weight dextrans purchased from the National Institute for the control of Pharmaceutical and Biological Products (Beijing, China) were used as standard. The changes in body weight of the various groups were recorded once a week. The rats were individually housed in metabolic cages for 24 h for urine collection at the end of the 2nd, 4th, 6th, 8th and 10th weeks. Urine protein levels were analyzed using kits from Nanjing Jiancheng (NanJing JianCheng Bio Inst, Nanjing, China). Blood glucose levels were determined after fasting for 4 h by collecting 50 μL blood from the rat’s eye socket at the end of the 2nd, 4th, 6th, 8th and 10th weeks. The blood glucose levels were determined using an enzymatic colorimetric assay [41]. At the end of the experiment, the rats were fasted overnight for 12 h, blood samples were collected from the aorta abdominalis and the serum was separated for measurement of the biochemical parameters. Scr (serum creatinine) and BUN (blood urea nitrogen) were analyzed with an Olympus Au640 (Tianjin, China) automatic biochemical analyzer. A radioimmunoassay was used to assay microalbumin, β2-microglobulin (β2-MG), serum insulin and glycosylated hemoglobin. 

### 3.6. Histopathological Procedures

After blood was collected, the rats were killed, the left kidneys rapidly removed and weighed, and tissue fragments fixed in 10% neutral buffered formalin solution, embedded in paraffin and then stained with hematoxylin and eosin (H&E). Renal pathological changes were observed under an optical microscope, and the glomerular diameter and the area were calculated.

### 3.7. Data Statistical Analysis

The data are presented as mean values ± 1 SD (*n* = 8–10). The data were analyzed with a one-way ANOVA, a Duncan’s multiple-range test and an LSD test at a significance level of *p* < 0.05. SPSS 17.0 software was used for the analysis.

## 4. Conclusions

In summary, our study demonstrated that FPS showed protective properties in DN rats. The most likely mechanism of renal protection by FPS is that FPS modulates metabolic abnormalities and reduces blood glucose levels. The results suggest that FPS can be considered as a potential candidate for developing a new anti-diabetic agent.
